# Development of a Scale-up Tool for Pervaporation Processes

**DOI:** 10.3390/membranes8010004

**Published:** 2018-01-15

**Authors:** Holger Thiess, Axel Schmidt, Jochen Strube

**Affiliations:** Institute for Separation and Process Technology, Clausthal University of Technology, Leibnizstraße 15, 38678 Clausthal-Zellerfeld, Germany; thiess@itv.tu-clausthal.de (H.T.); schmidt@itv.tu-clausthal.de (A.S.)

**Keywords:** pervaporation, physico-chemical modelling, up-scale, dehydration, ethanol, ethyl acetate

## Abstract

In this study, an engineering tool for the design and optimization of pervaporation processes is developed based on physico-chemical modelling coupled with laboratory/mini-plant experiments. The model incorporates the solution-diffusion-mechanism, polarization effects (concentration and temperature), axial dispersion, pressure drop and the temperature drop in the feed channel due to vaporization of the permeating components. The permeance, being the key model parameter, was determined via dehydration experiments on a mini-plant scale for the binary mixtures ethanol/water and ethyl acetate/water. A second set of experimental data was utilized for the validation of the model for two chemical systems. The industrially relevant ternary mixture, ethanol/ethyl acetate/water, was investigated close to its azeotropic point and compared to a simulation conducted with the determined binary permeance data. Experimental and simulation data proved to agree very well for the investigated process conditions. In order to test the scalability of the developed engineering tool, large-scale data from an industrial pervaporation plant used for the dehydration of ethanol was compared to a process simulation conducted with the validated physico-chemical model. Since the membranes employed in both mini-plant and industrial scale were of the same type, the permeance data could be transferred. The comparison of the measured and simulated data proved the scalability of the derived model.

## 1. Introduction

Extractive, pressure-swing and azeotropic distillation are typically applied as state-of-the-art unit operations for the separation of azeotropic mixtures, despite their technical complexity and large energy consumption [1,2]. Optimizing the energy efficiency of new and established processes becomes more and more important in a globalized market [3]. Hence, pervaporation and vapour permeation processes were developed as stand-alone or hybrid processes as alternatives to distillation unit operations. Pervaporation is a unit operation through which a liquid mixture is selectively separated by a membrane. The driving force of the process is the partial pressure difference between the feed and permeate side of the membrane, instead of the difference in volatility on which distillation is based. Thereby, a pervaporation process is able to separate azeotropic mixtures without the use of entrainers or a variation in pressure [4].

The design and optimization of pervaporation processes as well as the possible interconnection with other unit operations necessitates a high theoretical understanding of the process, membrane and feed mixture, thus leading to a demand for proper engineering tools for designing pervaporation unit operations.

The scope of this work is the development of a physico-chemical model which, if combined with experiments for the model parameter determination, can be used as an engineering tool for the scale-up and optimization of pervaporation processes. The physico-chemical model includes balances for mass, enthalpy and impulse. It incorporates the transport phenomena known in literature such as the solution-diffusion mechanism, concentration and temperature polarization, axial dispersion and pressure drop in open channels as well. In order to determine the permeance and to validate the model, dehydration experiments were conducted with the two binary chemical systems ethanol/water and ethyl acetate/water. The experimental design incorporates variations in mixture composition, feed temperature, permeate pressure and feed volume flow. The ternary system ethanol/ethyl acetate/water was investigated at two temperatures for a feed composition close to the azeotropic point as well.

With the derived binary permeance data, simulations are compared to experimental data of the industrially relevant ternary mixture ethanol/ethyl acetate/water. The scalability of the physico-chemical model was investigated by comparing data from an industrial pervaporation plant (200 m^2^, ethanol/water) with the simulation of the derived model and permeance data.

## 2. Theory

In order to derive a consistent physico-chemical model, a defined control volume combined with balancing equations describing mass, heat and impulse transfer is essential. In the following the fundamental equations used for mass, enthalpy and momentum balancing as well as the model parameter determination methods are introduced.

### 2.1. Mass Transfer

For the characterization of the mass transfer of a pervaporation process from the feed solution to the permeate or retentate respectively, three main effects have to be described. The mass transfer through the feed channel of the membrane module can be described using the distributed plug-flow model. The selective transport through a dense membrane is commonly depicted by the solution-diffusion model. The third main effect is the concentration polarization resulting from the formed boundary layer on the membrane’s surface.

#### 2.1.1. The Solution-Diffusion Model

The following derivation of the solution-diffusion model is based on the work of Wijmans and Baker [5].Fundamental for the mathematical formulation of the permeate flux by the solution-diffusion model, is the thermodynamic description of the driving force by the gradient of the chemical potential [5]. The flux $J_{i}$ of a component can be expressed by Equation (1)

(1) $$J_{i}=-L_{i}\frac{d\mu_{i}}{dz},$$

with $L_{i}$ being a proportionality coefficient and $\mu_{i}$ the chemical potential. Restricting to molar concentration and pressure gradients, the chemical potential is written as [5]:

(2) $$d\mu_{i}=RT\;dln\left(\gamma_{i}x_{i}\right)+v_{i}\;dp,$$

with the gas constant R, the temperature T, the component’s activity coefficient $\gamma_{i}$, the molar fraction $x_{i}$, the molar volume $v_{i}$ and the pressure p. For a dense membrane, the pressure inside the membrane is constant and equal to the pressure in the feed channel. Under this premise combining Equations (1) and (2) results in Equation (3)

(3) $$J_{i}=-L_{i}\;RT\;\frac{dln\left(\gamma_{i}x_{i}\right)}{dz}=-L_{i}\frac{RT}{x_{i}}\;\frac{dx_{i}}{dz}.$$

Similar to Fick’s law the proportionality term is replaced by the diffusion coefficient $D_{i}$ (Equation (4)), resulting in Equation (5).

(4) $$D_{i}=L_{i}\frac{RT}{x_{i}}$$

(5) $$J_{i}=D_{i}\;\frac{x_{i_{0\left(m\right)}}-x_{i_{l\left(m\right)}}}{l}$$

Therefore, the difference in molar concentration over the membrane thickness has to be investigated in further detail. The integration of Equation (2), while using the vapour pressure $p_{i_{sat}}$ as pressure reference, results in Equation (6) for incompressible and Equation (7) for compressible fluids, respectively.

(6) $$\mu_{i}=\mu_{i}^{0}+RT\;ln\left(\gamma_{i}x_{i}\right)+v_{i}\left(p-p_{i_{sat}}\right)$$

(7) $$\mu_{i}=\mu_{i}^{0}+RT\;\ln\left(\gamma_{i}x_{i}\right)+RT\;\ln\left(\frac{p}{p_{i_{sat}}}\right)$$

The following two assumptions are made [5]:

1. diffusion through the membrane being the rate-determining step,
2. equilibrium between both phases at feed- and permeate-side membrane surface, resulting in Equations (8) and (9).
  (8) $$\mu_{i_{0}}=\mu_{i_{0\left(m\right)}}$$
  (9) $$\mu_{i_{l\left(m\right)}}=\mu_{i_{l}}$$

The feed side of the membrane can thus be expressed by Equation (10)

(10) $$\mu_{i}^{0}+RT\;\ln\left(\gamma_{i_{0}}^{L}x_{i_{0}}^{L}\right)+v_{i}\;(p_{0}-p_{i_{sat}})=\mu_{i}^{0}+RT\;\ln\left(\gamma_{i_{0\left(m\right)}}x_{i_{0\left(m\right)}}\right)+v_{i}\;(p_{0}-p_{i_{sat}}),$$

which is simplified to Equation (11)

(11) $$x_{i_{0\left(m\right)}}=\frac{\gamma_{i_{0}}^{L}x_{i_{0}}^{L}}{\gamma_{i_{0\left(m\right)}}}=K_{i}\cdot x_{i_{0}}^{L},$$

where $K_{i}$ is the liquid phase sorption coefficient. In analogy to the retentate, the permeate side is expressed with Equation (12):

(12) $$\mu_{i}^{0}+RT\;ln\left(\gamma_{i_{l}}^{G}x_{i_{l}}^{G}\right)+RT\;ln\left(\frac{p_{l}}{p_{i_{sat}}}\right)=\mu_{i}^{0}+RT\;ln\left(\gamma_{i_{l\left(m\right)}}x_{i_{l\left(m\right)}}\right)+v_{i}\;(p_{0}-p_{i_{sat}}).$$

**Figure 1:**
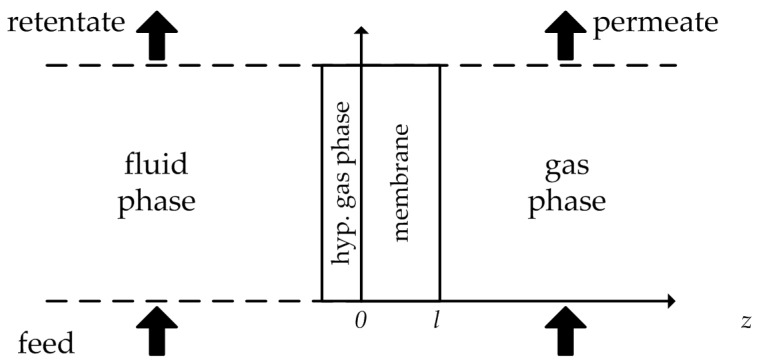
Hypothetical gas phase between feed solution and membrane. Illustration adapted from [6].

Rearranging Equation (13) leads to

(13) $$x_{i_{l\left(m\right)}}=\frac{\gamma_{i_{l}}^{G}}{\gamma_{i_{l\left(m\right)}}}\cdot \frac{p_{l}}{p_{i_{sat}}}\cdot x_{i_{l}}^{G}\cdot exp\left(\frac{-v_{i}\left(p_{0}-p_{i_{sat}}\right)}{RT}\right),$$

where the exponential term is near one, thus resulting in Equation (14)

(14) $$x_{i_{l\left(m\right)}}=\frac{\gamma_{i_{l}}^{G}}{\gamma_{i_{l\left(m\right)}}}\cdot \frac{p_{i_{l}}}{p_{i_{sat}}}=K_{i}^{G}\cdot p_{i_{l}},$$

with the gas phase sorption coefficient $K_{i}^{G}$. In order to work with just one type of sorption coefficient, a hypothetical gas phase between the liquid phase and the membrane is introduced (Figure 1).

Thereby, the equilibrium between the liquid and the hypothetical gas phase can be expressed by Equation (15)

(15) $$\mu_{i}^{0}+RT\;ln\left(\gamma_{i_{0}}^{L}\;x_{i_{0}}^{L}\right)+v_{i}\;\left(p_{0}-p_{i_{sat}}\right)=\mu_{i}^{0}+RT\;ln\left(\gamma_{i_{0}}^{G}\;x_{i_{0}}^{G}\right)+RTln\left(\frac{p_{0}}{P_{i_{sat}}}\right),$$

where rearranging leads to Equation (16)

(16) $$x_{i_{0}}=\frac{\gamma_{i_{0}}^{G}}{\gamma_{i_{0}}^{L}\cdot p_{i_{sat}}}p_{i_{0}}$$

Implementing Equation (16) in Equation (11) results in

(17) $$x_{i_{0\left(m\right)}}=\frac{\gamma_{i_{0}}^{G}}{\gamma_{i_{0\left(m\right)}}}\cdot \frac{p_{i_{0}}}{p_{i_{sat}}}=K_{i}^{G}\cdot p_{i_{0}},$$

where the gas sorption coefficient is used as well. Incorporating Equations (14) and (17) into Equation (5) results in

(18) $$J_{i}=D_{i}\;K_{i}^{G}\cdot \frac{p_{i_{0}}-p_{i_{l}}}{l}=P_{i}^{G}\cdot \frac{p_{i_{0}}-p_{i_{l}}}{l},$$

where in the product $D_{i}\;K_{i}^{G}$ can be expressed as the permeability $P_{i}^{G}$ and the driving force is reduced to the partial pressure difference between both sides of the membrane. The partial pressures can be rephrased using Equations (19) and (20). The ratio between the permeability and the membrane thickness is defined as the Permeance $Q_{i}$, since the exact dimensions of the active membrane layer are often not accessible [7]. Implementing Equations (19)–(21) into Equation (18) results in the transport Equation (22), usable to calculate the permeate flux. It becomes obvious that the flux is dependent on the feed concentration, feed temperature and the permeate pressure.

(19) $$\mu_{i_{0}}=\mu_{i_{0\left(m\right)}}$$

(20) $$\mu_{i_{l\left(m\right)}}=\mu_{i_{l}}$$

(21) $$\mu_{i_{0}}=\mu_{i_{0\left(m\right)}}$$

(22) $$J_{i}=Q_{i}\cdot \left({p_{i}}_{sat}\cdot x_{i_{0}}^{L}\cdot \gamma_{i_{0}}^{L}-p_{p}\cdot y_{i_{l}}\right)$$

#### 2.1.2. Determination of the Permeance

In literature, several approaches for the determination of the permeance $Q_{i}$ exist. Since the effects sorption and diffusion are dependent on concentration and temperature, it becomes clear, that the permeance must show this dependency as well. For short-cut models, describing isothermal processes without a larger change in concentration, the permeance can be assumed constant (Equation (23)), to obtain a quick prediction of the flux with just few experiments [7].

(23) $$Q_{i}=constant$$

For non-steady-state and/or non-isothermal conditions, other approaches have to be used. To describe the kinetics of sorption and diffusion depending on the temperature, the Arrhenius approximation in Equation (24) is commonly used in literature [8].

(24) $$Q_{i}=Q_{i}^{0}\cdot exp\left(-\frac{B_{i}}{R}\;\left(\frac{1}{T_{0}}-\frac{1}{T}\right)\right)$$

The reference permeance $Q_{i}^{0}$ and the permeance parameter $B_{i}$, which is in literature often referred to as the activation energy in literature [2], based on the reference temperature $T_{0}$ are pseudo-physical parameters, which can be determined using experimental data. In order to implement a concentration dependency, different approaches are known, based on the applied membranes and solvents. Equations (25) and (26) show different modifications of the Arrhenius approach, to incorporate the water concentration’s influence [2,8] on the permeance for hydrophilic membranes.

(25) $$Q_{i}=Q_{i}^{0}\cdot exp\left(A_{i}w_{H_{2}O}-\frac{B_{i}}{R}\;\left(\frac{1}{T_{0}}-\frac{1}{T}\right)\right)$$

(26) $$Q_{i}=Q_{i}^{0}\cdot w_{H_{2}O}^{A_{i}}\cdot exp\left(-\frac{B_{i}}{R}\;\left(\frac{1}{T_{0}}-\frac{1}{T}\right)\right)$$

In some cases, no temperature dependency for the permeance is observed. This indicates a compensation between the exothermal sorption and the acceleration of the diffusion process, resulting in a temperature-independent permeance [9].

By integrating the diffusion and solubility coefficient into the permeance, all coupling phenomena based either on solubility or diffusivity of the components are merged. The utilization of the Arrhenius approach and the structure of Equations (24)–(26) identify the determination of the permeance as clearly semi-empirical.

The ability to separate a fluid mixture is commonly described using separation factors or the selectivity. The binary selectivity of a pervaporation process is defined in Equation (27) by the permeance ratio of the more permeable component over the less permeable [10]:

(27) $$\alpha_{ij}=\frac{Q_{i}}{Q_{j}}.$$

#### 2.1.3. Concentration Polarization

**Figure 2:**
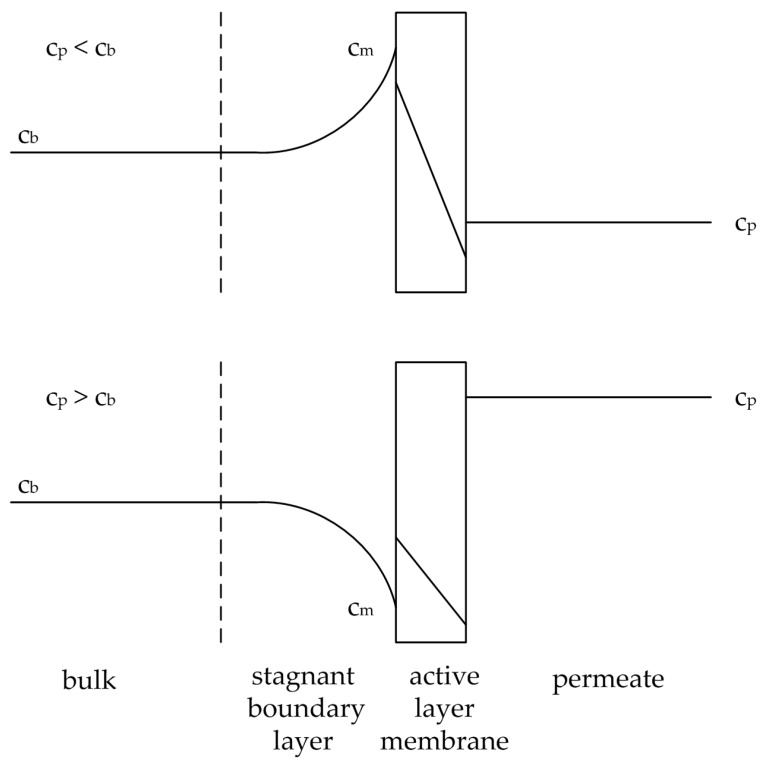
Illustration of the concentration polarization effect for an accumulating substance (above) and a depleting substance (below). Adapted from [17].

As described by the film theory the flow velocity at the membrane surface becomes zero and a boundary layer is formed [11]. The driving force of the membrane process causes a convective transport of the bulk to the boundary layer. The permeate flux, based on the permeating components leaves the boundary layer (Figure 2). The retained component enriches at the membrane surface, while the permeating component depletes. Hence, the concentration gradient between the membrane surface and the bulk induces diffusion, either in the direction of the bulk for the retained component or to the membrane surface for the permeating component. Balancing the boundary layer leads to Equation (28), where the terms are substituted by Equations (29)–(31),

(28) $$J_{i,Konv}+J_{i,\;Diff}=J_{i,TM},$$

(29) $$J_{i,\;Konv}=J_{total}\cdot w_{i},$$

(30) $$J_{i,\;Diff}=-\rho_{total,F}\cdot D_{i}\;\frac{dw_{i}}{dz},$$

(31) $$J_{i,\;TM}=J_{total}\cdot w_{i,P},$$

with the diffusion coefficient $D_{i}$, the the feed mixture density $\rho_{tot,f}$ and the total permeate flux $J_{tot}$

(32) $$J_{tot}\cdot w_{i}+\rho_{tot,f}\cdot D_{i}\;\frac{dw_{i}}{dz}=J_{tot}\cdot w_{i,P}$$

Integrating Equation (32) with the given boundaries shown in Equation (33) and rearranging leads to the concentration polarization Equation (34).

(33) $$\int_{w_{i}=w_{i,fm}}^{w_{i}=w_{i,f}}\frac{dw_{i}}{w_{i}-w_{i,p}}=-\int_{z=0}^{z=\delta }\frac{J_{tot}}{\rho_{tot,f}\cdot D_{i}}\;dz$$

(34) $$J_{tot}=\rho_{tot,f}\;\frac{D_{i}}{\delta }ln\left(\frac{w_{i,fm}-w_{i,p}}{w_{i,f}-w_{i,p}}\right)$$

The indices FM, F and P stand for the membrane surface on the feed-side, the bulk and the permeate respectively. The ratio of the diffusion coefficient to the boundary layer thickness δ can be superseded by the mass transfer coefficient $k_{i}$ (Equation (35)).

(35) $$k_{i}=\frac{D_{i}}{\delta }$$

The mass transfer coefficient may be calculated by the *Sh*-correlation shown in Equation (36), based on the dimensionless quantities *Re* (Reynolds number), *Sc* (Schmidt number) and Sh (Sherwood number). The definitions of the *Re* and *Sc* are given in Equations (37) and (38). The parameters a, b, c and d are listed on literature based on different membrane modules and flow regimes [12,13,14,15,16].

(36) $$Sh=\frac{k\;d_{h}}{D_{i}}=a_{1}\cdot Re^{a_{2}}\cdot Sc^{a_{3}}\cdot {\left(\frac{d_{h}}{l}\right)}^{a_{4}}$$

(37) $$Re=\frac{\rho \;u\;d_{h}}{\eta }$$

(38) $$Sc=\frac{\eta }{\rho \cdot D}$$

The concentration polarization influences the flux for pervaporation processes in two ways. The more retained component accumulates at the membrane surface, thereby increasing its partial pressure difference (see solution-diffusion model), hence its permeate flux. The less retained component depletes at the membrane surface, resulting in a smaller permeate flux. This phenomenon is shown in Figure 2. Both effects lead to a worse selectivity of the pervaporation process. An increase of the mass transfer coefficients hones the diffusional transport and hence the selectivity of the process.

#### 2.1.4. The Distributed Plug Flow Model

The mass transfer in the feed channel of the membrane module can be described by the distributed plug flow (DPF) model [18]. The DPF model considers convective and dispersive mass transfer to and from the control volume. Additionally, the accumulation of a component in the given control volume and the mass transfer leaving the control volume due to the permeate flux are taken into account [19]. Balancing all these phenomena results in the mass balance shown in Equation (39).

(39) $$\frac{\partial m_{i}}{\partial t}={{\dot{m}}_{i,conv}|}_{z}-{{\dot{m}}_{i,conv}|}_{z+dz}+{{\dot{m}}_{i,disp}|}_{z}-{{\dot{m}}_{i,disp}|}_{z+dz}-{\dot{m}}_{i,PV}$$

Each term can be rewritten, as shown in Equations (40)–(43):

(40) $$\frac{\partial m_{i}}{\partial t}=\frac{\partial }{\partial t}\left(c_{i}\cdot dV\right)=\frac{\partial }{\partial t}\left(c_{i}\cdot A_{Q}\cdot dz\right)=\frac{\partial c_{i}}{\partial t}\cdot A_{Q}\cdot dz.$$

(41) $${{\dot{m}}_{i,conv}|}_{z}-{{\dot{m}}_{i,conv}|}_{z+dz}\approx -\frac{\partial {\dot{m}}_{i,conv}}{\partial z}\cdot dz=-u\cdot A_{Q}\cdot \frac{\partial c_{i}}{\partial z}\cdot dz$$

(42) $${{\dot{m}}_{i,disp}|}_{z}-{{\dot{m}}_{i,disp}|}_{z+dz}\approx -\frac{\partial {\dot{m}}_{i,disp}}{\partial z}\cdot dz=\frac{\partial }{\partial z}\;\left(A_{Q}\cdot D_{ax,i}\cdot \frac{\partial c_{i}}{\partial z}\right)\cdot dz=A_{Q}\cdot D_{ax,i}\cdot \frac{\partial^{2}c_{i}}{\partial z^{2}}\cdot dz$$

(43) $${\dot{m}}_{i,PV}=\frac{\partial {\dot{m}}_{i,PV}}{\partial z}\cdot dz=J_{i}\cdot dA=J_{i}\cdot w_{m}\cdot dz.$$

In order to work with mass fractions, Equations (40)–(43) are set against Equation (44) resulting in Equation (45).

(44) $${m_{tot}|}_{z+dz}=\rho_{tot}\cdot dV=\rho_{tot}\cdot A_{Q}\cdot dz=\rho_{tot}\cdot \left(w_{m}\;h_{ch}\right)\cdot dz$$

(45) $$\frac{\partial w_{i}}{\partial t}=-u\cdot \frac{\partial w_{i}}{\partial z}+D_{ax,i}\cdot \frac{\partial^{2}w_{i}}{\partial z^{2}}-\frac{J_{i}}{\rho_{tot}\cdot h_{ch}}$$

### 2.2. Heat Transfer

The phase change of the permeate makes the pervaporation process unique compared to other common membrane processes, elucidating on the other hand, that an enthalpy balance incorporating possible polarization effects is needed.

#### 2.2.1. Enthalpy Balance

The enthalpy balance of a pervaporation module includes the enthalpy flow of the feed, the enthalpy flow of the permeate leaving the feed channel and the resulting retentate enthalpy flow. Due to permeate flux and the vaporization of the permeate, the retentate enthalpy flow reduces over the membrane length [20]. The overall enthalpy balance for the module is:

(46) $${\dot{H}}_{f}={\dot{H}}_{r}+{\dot{H}}_{p}$$

The enthalpy flow of the feed solution can be derived by the mass flow, the specific heat capacity of the feed as well as its temperature (Equation (47)):

(47) $${\dot{H}}_{f}={\dot{m}}_{f}\cdot {\tilde{c}}_{p,f}\;\left(T_{F}-T_{0}\right).$$

With the simplification that the temperature in feed and permeate is the same, Equation (48) shows that the enthalpy flow of the permeate is based on the permeate flux, the membrane area and the average specific enthalpy of the permeate.

(48) $${\dot{H}}_{p}={\tilde{h}}_{p}^{G}\cdot J_{tot}\cdot A_{m}={\tilde{h}}_{p}^{G}\cdot J_{tot}\cdot w_{m}\cdot l_{m}$$

To describe the change in enthalpy over the length ∆z, Equation (49) is derived:

(49) $${{\dot{m}}_{f}\cdot {\tilde{c}}_{p,f}\;\left(T_{f}-T_{0}\right)|}_{z}={{\dot{m}}_{f}\cdot {\tilde{c}}_{p,f}\;\left(T_{f}-T_{0}\right)|}_{z+∆z}+{{\tilde{h}}_{p}^{G}\cdot J_{tot}\cdot w_{m}\cdot ∆z|}_{z}.$$

The differential change in the enthalpy flow for feed- and permeate-side of the membrane can thereby be determined by:

(50) $$\frac{d{\dot{H}}_{r}\left(z\right)}{dz}=-J_{tot}\left(z\right)\cdot w_{m}\cdot {\tilde{h}}_{p}^{G},$$

(51) $$\frac{d{\dot{H}}_{p}\left(z\right)}{dz}=J_{tot}\left(z\right)\cdot w_{m}\cdot {\tilde{h}}_{p}^{G}.$$

#### 2.2.2. Temperature Polarization

**Figure 3:**
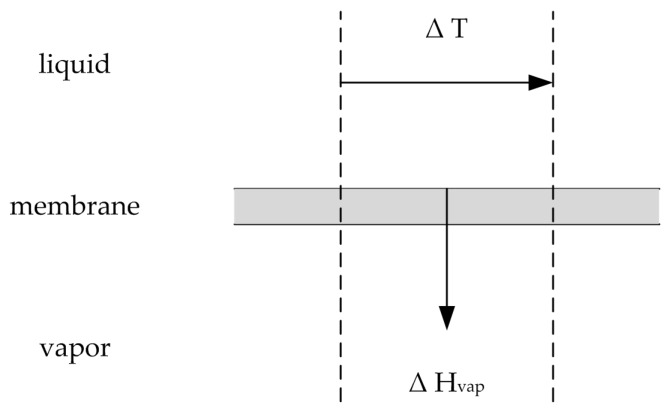
Illustration of the heat transfer via vaporization. Adapted from [21].

During the pervaporation process, the permeating components desorb and vaporize on the permeate side of the membrane. Thus, vaporization enthalpy $∆{\tilde{h}}_{i}^{V}$ is needed and withdrawn from the feed solution (see Figure 3). The overall vaporization enthalpy required is the enthalpy difference between the gaseous and liquid phase, shown in Equation (52).

(52) $$∆{\tilde{h}}_{tot}^{V}={\tilde{h}}_{p}^{G}-{\tilde{h}}_{p}^{L}$$

Similar to the effect of concentration polarization the developed boundary layer on the membrane surface causes a temperature gradient between the bulk and the membrane surface on the feed side. Hence, similar to concentration polarization the effect of temperature polarization decreases the driving force and thereby the permeate flux. The enthalpy flow needed to vaporize the permeate mass flow ${\dot{H}}_{vap}$ is connected to the induced temperature gradient by the heat transfer approach shown in Equations (53) and (54) [21]:

(53) $${\dot{H}}_{vap}=J_{tot}\cdot A\cdot ∆{\tilde{h}}_{tot}^{vap}=J_{tot}\cdot A\cdot \left({\tilde{h}}_{p}^{G}-{\tilde{h}}_{p}^{L}\right),$$

(54) $$\frac{{\dot{H}}_{vap}}{A_{m}}=\frac{\lambda }{\delta }\left(T_{f}-T_{fm}\right).$$

Analogous to Section 2.1.3 the thermal conductivity coefficient and boundary layer thickness are combined into the heat transfer coefficient, given by Equation (55)

(55) $$\alpha =\frac{\lambda }{\delta }$$

The Sherwood correlation introduced in Section 2.1.3 is based on the Nusselt correlation for heat transfer. Sherwood and Schmidt number represent the mass transfer analogies to Nusselt and Prandtl number in heat transfer. The definitions of the *Nu* and *Pr* are given by Equations (56) and (57).

(56) $$Nu=\frac{\alpha \cdot d_{h}}{\lambda }=a_{1}\cdot Re^{a_{2}}\cdot Pr^{a_{3}}\cdot {\left(\frac{d_{h}}{l}\right)}^{a_{4}}$$

(57) $$Pr=\frac{\eta \cdot {\tilde{c}}_{p}}{\lambda \cdot M}$$

### 2.3. Impulse Transfer

The pressure drops across the feed and permeate channels is of importance for a pervaporation process. If the pressure on the feed side decreases below the saturated vapour pressure of a component, the component will vaporize. A low permeate pressure plays a key role in the driving force of the solution-diffusion mechanism. Hence, the pressure drop on the permeate side must to be minimized. Larger permeate fluxes and long distances between membrane and vacuum pump aggravate this task [5,22]. In order to describe the pressure, drop on the feed- and permeate-side, the phase and flow regime have to be known. The pressure drop in channels can be expressed by the Darcy-Weisbach-equation:

(58) $$∆p=\zeta \;\frac{l}{d_{h}}\;\frac{\rho \;u^{2}}{2}.$$

Rearranging Equation (58) to describe the differential change in pressure drop results in:

(59) $$\frac{dp}{dz}=-\zeta \;\frac{\rho \;u^{2}}{2\;d_{h}}.$$

Depending on the flow regime, the drag coefficient ζ for open channels in plate and frame modules can be expressed by Equation (60) for laminar flow (Re<2320) and Equation (61) for turbulent flow (Re>2320) [21].

(60) $$\zeta =\frac{38}{Re}$$

(61) $$\zeta =\frac{1.22}{Re^{0.252}}$$

Implementing Equation (60) for laminar flow and Equation (61) for turbulent flow into Equation (58) leads to:

(62) $$\frac{dp}{dz}=-19\;\eta \frac{u}{d_{h}^{2}}$$

(63) $$\frac{dp}{dz}=-0.61\;\eta^{0.252}\frac{\rho^{0.748}u^{1.748}}{d_{h}^{1.252}}.$$

Since the feed solution can be considered as an incompressible fluid, the velocity can be expressed with the continuity equation for both laminar and turbulent flow regime:

(64) $$\frac{dp_{f}}{dz}=-19\;\eta_{F}\frac{{\dot{V}}_{f}}{d_{h,f}^{2}w_{m}h_{ch,f}}$$

(65) $$\frac{dp_{f}}{dz}=-0.61\;\eta_{f}^{0.252}\frac{\rho_{f}^{0.748}{\dot{V}}_{f}^{1.748}}{d_{h,f}^{1.252}w_{m}^{1.748}h_{ch,f}^{1.748}}.$$

In case of the vapour on the permeate side, the ideal gas law is utilized since the permeate pressure is adequately low, shown in Equation (66).

(66) $$u_{P}=\frac{R\;T_{P}\;{\dot{n}}_{p}}{p_{p}\;A_{Q}}=\frac{R\;T_{p}\;{\dot{n}}_{p}}{p_{p}\;w_{m}\;h_{ch,p}}$$

Implementation of Equation (66) in Equations (62) and (63) leads to Equations (67) and (68) which are used to describe the discretized pressure drop of the permeate for laminar and turbulent flow regimes.

(67) $$\frac{dp_{p}}{dz}=-19\;\eta_{p}\;\frac{R\;T_{p}\;{\dot{n}}_{p}}{p_{p}\;w_{m}\;h_{ch,p}\;d_{h,p}^{2}}$$

(68) $$\frac{dp_{p}}{dz}=-0.61\;\eta_{p}^{0.252}\;\rho_{p}^{0.748}\;\frac{{\left(\frac{R\;T_{p}\;{\dot{n}}_{P}}{p_{p}\;w_{m}\;h_{ch,p}}\right)}^{1.748}}{\;d_{h,p}^{1.252}}$$

For the sake of completeness, it has to be stated that between *Re* 2000 and *Re* 4000 a transition state exists and even within the turbulent flow regime the nature of the flow can be further divided.

## 3. Materials and Methods

### 3.1. The Rigorous Model

The differential equations of the pervaporation model are solved through orthogonal collocation on Jacobi-polynomial basis and integration based on an incremental- and order-controlled gear algorithm. The investigated pervaporation processes were batch processes. Therefore, the model consists of three submodels: the feed tank, the membrane module and a permeate tank representing the condensed permeate. In the pervaporation plants as well as the model, the feed leaves the feed tank and enters the module. The permeate stream leaves the module and accumulates in the permeate tank. The retentate stream is redirected into the feed tank. In both feed and permeate tank a simple mass balance was implemented. The initial mass of each component, the temperature and the overall feed mass flow and the pressure are needed for the feed tank submodel. The permeate tank submodel requires the permeate pressure. Both, feed and permeate tank are assumed to be ideally mixed. The initial masses of the components in the permeate tank are zero.

**Figure 4:**
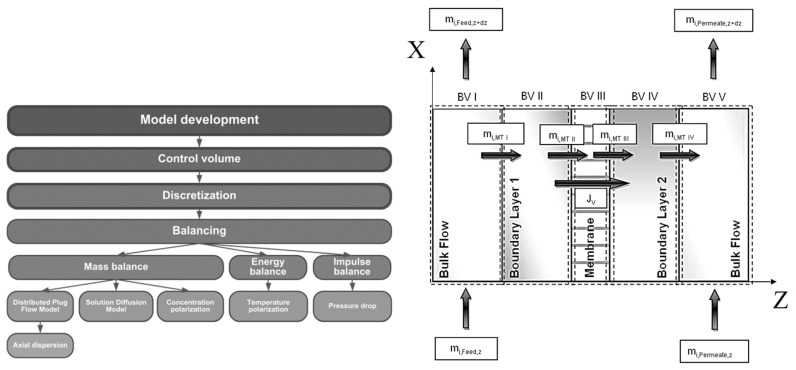
**Left**: Course of action used for the pervaporation model development. **Right**: Overview of the five control volumes used in modelling and simulation. Adapted from [23].

Figure 4 depicts the development of the rigorous pervaporation model used for the membrane module in this work. Based on the shown control volume the model includes balances for mass, enthalpy and impulse. The overall control volume is divided into five sub-control volumes. CV1 is the feed channel of the membrane module without the boundary layer on the membrane surface.

The mass balance to describe the bulk flow is based on the DPF model (Equation (45)). As boundary conditions for entrance and exit of the feed channel Equations (69) and (70) were implemented:

(69) $${w_{r,i}|}_{Z=0}=\frac{{\dot{m}}_{f,i}}{{\dot{m}}_{f,tot}},$$

(70) $${\frac{dw_{r,i}}{dz}|}_{z=L}=0.$$

Equation (50) was utilized to describe the enthalpy balance in the feed channel and its exit in CV1. The entrance boundary condition of the feed channel is based on the feed temperature, the feed composition and the feed mass flow:

(71) $${{\dot{H}}_{r}|}_{Z=0}={\dot{H}}_{f}={\dot{m}}_{f,tot}\cdot {\tilde{c}}_{p,f}\;\left(T_{f}-T_{0}\right).$$

As impulse balance in CV1 Equation (64) combined with Equations (72) and (73) were incorporated into the model.

(72) $${p_{r}|}_{z=0}=p_{f}$$

(73) $${\frac{dp_{r}}{dz}|}_{z=L}=0$$

CV2 represents the boundary layer on the feed side of the membrane. Concentration and temperature polarization effects occurring in CV2 were implemented with Equations (34) and (54) respectively. The mass transfer through the membrane in CV3 was described by the LDM (Equation (22)). CV4 was neglected in this model. Equation (67) coupled with the boundary conditions shown in Equations (74) and (75) were used to describe the pressure drop in CV5:

(74) $${\frac{dp_{p}}{dz}|}_{z=0}=0,$$

(75) $${p_{p}|}_{z=L}=p_{p\_exp}$$

For the description of the mass transfer in CV5, a simple balancing of the streams was (Equation (76)) used together with the boundary condition shown in Equation (77):

(76) $$\frac{d{\dot{m}}_{p,i}}{dz}=J_{i}\cdot w_{m},$$

(77) $${{\dot{m}}_{p,i}|}_{z=0}=0$$

### 3.2. Model Parameter Determination

Material data such as activity coefficients, vapour pressures, diffusion coefficients, thermal conductivity coefficients, the average density, dynamic viscosity, molecular weight, molar enthalpies and the molar heat capacity of the fluid were calculated with Aspen Properties^™^ (AP, Calgary, AB, Canada). Via call-functions AP was connected to the pervaporation model. NRTL (Non-Random-Two-Liquids [24]) was chosen as the thermodynamic model.

The hydraulic diameter for a given channel is defined as four times the cross-sectional area divided by the wetted perimeter:

(78) $$d_{h}=4\frac{A_{Q}}{U_{wetted}}=2\frac{w_{m}\cdot h_{ch}}{w_{m}+h_{ch}}.$$

With the known material data and the hydraulic diameter, the Sherwood (Equation (36)) and Nusselt correlation (Equation (56)) were utilized to obtain the mass transfer and heat transfer coefficient. The required parameters $a_{1}$ to $a_{4}$ were obtained from literature data for the respective flow regime and listed in Table 1.

The axial dispersion coefficient $D_{ax}$ for Reynolds numbers smaller than 10,000 in open channels was correlated by Equation (79) [19]:

(79) $$\frac{D_{ax}}{uL}=\frac{1}{Re\cdot Sc}+\frac{Re\cdot Sc}{192}.$$

### 3.3. Experimental Work

All pervaporation experiments conducted for this study utilized the hydrophilic DeltaMem PERVAP™ 4101 membrane (DeltaMem AG (former Sulzer), Muttenz, Switzerland). The flatsheet membrane is a composite membrane consisting of an active layer of polyvinyl alcohol (PVA) on a support layer of polyacrylonitrile (PAN). The rectangular test cell had one open channel with a channel length of 28.2 cm. The resulting membrane area was 0.017 m^2^. The flow regime throughout the experiments were calculated to be laminar for the utilized ranges of feed volume flow.

In this study, the binary systems of ethanol/water and ethyl acetate/water—as well as the respective ternary system ethanol/ethyl acetate/water—were examined. Ethanol (VWR International, Randor, PA, USA) and ethyl acetate (Merck Millipore, Burlington, MA, USA) were supplied with a purity greater than 99.5%. The analysis of the organic solvents was done via gas chromatography with a VF-1ms column from Agilent (Santa Clara, CA, USA). The water analytics were conducted by Karl Fischer titration with a TitroLine from SI-Analytics (Mainz, Germany).

**Figure 5:**
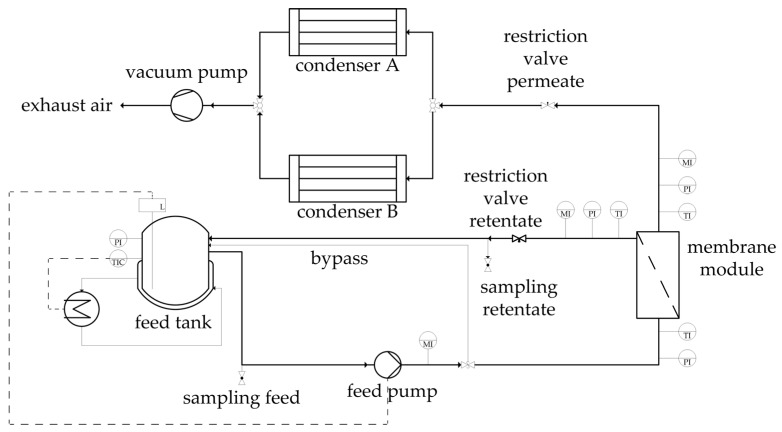
Flowsheet of the pervaporation unit utilized for the binary and ternary experiments.

Figure 5 depicts the flowsheet of the pervaporation unit used for the binary and ternary experiments. In each experiment, the starting feed solution was 1.5 kg. The feed was temperature-controlled via the jacketed feed tank. The solution was pumped into the membrane module. The retentate flow was redirected into the feed tank. By utilizing a vacuum pump the permeate flux was vaporized and transported to the cooling traps were the permeate condensed. Every hour, feed and retentate samples were taken and the cooling trap weighed and changed. After the cooling trap defrosted, a permeate sample was taken. All experiments were carried out for ten hours. Before each experiment was started, a preconditioning of the unit and membrane was performed with the experimental feed temperature with the permeate restriction valve closed. The experiments are partitioned in three groups: the dehydration of ethanol/water mixtures, of ethyl acetate/water mixtures and of an ethanol/ethyl acetate/water mixture. The two binary systems utilized a similar experimental design based on Design of Experiments (DoE), where the feed temperature, the feed composition, the permeate pressure and the feed volume flow were varied. The reproducibility of both systems was investigated by thrice-conducted centre point experiments. All binary experiments were conducted at a feed pressure of 3.5 bar, leading to boiling points above 110 °C for all investigated binary feed compositions. This ensures the liquid state of the feed solution. The ternary system was investigated for two temperatures. For each experimental set one membrane was utilized for all respective experiments. To investigate a possible change in the dehydration performance over the course of the experimental design, a centre point experiment was conducted near the beginning, in the middle and at the end of each experimental plan. The experimental design is listed in Table 2 for ethanol/water, in Table 3 for ethyl acetate/water and in Table 4 for the ternary system. The experimental design of the binary system experiments was subdivided into two data sets. The first data set was used for the determination of the permeance function and its parameters. The second data set on the other hand conduced the validation of the physico-chemical model. The determination of the permeance parameters for each component ($Q_{i}^{0}$, $A_{i}$, $B_{i}$) was done by a nonlinear least squares regression, minimizing the weighted square error between simulation and experimental results for the feed mass fraction and the permeate flux. The NL2SOL was used as solver [25]. The initial values were taken from permeance data obtained in preliminary tests. These initial values were varied as well to make sure to find the true minima of the weighted square error. Table 2 and Table 3 highlight the experiments deployed for the permeance determination. With the derived permeance data, the simulation results were compared to the experimental data for the remaining experiments in order to validate the physico-chemical model along with the employed model parameters.

To investigate the ternary system, a feed mixture close to the azeotropic point was chosen. The composition of the azeotropic mixture was calculated with AP utilizing the Property model NRTL for the vapour-liquid-liquid phases. At 1.01325 bar the azeotropic mixture contains 13.75% ethanol, 77.9% ethyl acetate and 8.35% water (mass fractions), boiling at 70.5 °C. At the investigated feed pressure of 5 bar, the boiling point of the azeotropic mixture is 122 °C.

In order to test the scalability of the physico-chemical model large scale data from an industrial pervaporation plant was compared to the respective simulation. The membrane module of the pervaporation plant utilized flat-sheet membranes with a total area of 50 m^2^. In total four of these membrane modules were combined achieving a total membrane area of 200 m^2^. The feed stream is divided onto two modules (parallel array). After each of these modules the retentate stream is heated-up to feed solution conditions and enters a second membrane module (series array). This module configuration was adapted for the membrane module submodel. 15,000 kg of ethanol/water feed solution were dehydrated for 24 h with the pervaporation plant with samples taken every 2 h. The same membrane type was used for the industrial plant as in the experimental pervaporation unit. The flow regime is comparable to the lab scale module. Table 5 list the set process parameters utilized to generate the large-scale data.

## 4. Results

In all pervaporation experiments the mass balances were closed with lower than 7% deviation (4.1% averaged). The reproducibility was examined by three centre point experiments for both binary systems as well as the ternary system experiments at 95 °C, which were also conducted three times. The averaged relative standard deviation for ethanol/water (ethyl acetate/water) was 4.6% (6%) for the water mass fraction in the feed and 13% (28%) for the total permeate flux respectively. For the ternary system, the average relative standard deviation was 2.8% for the water mass fraction in the feed and 9.2% for the total permeate flux. The averaged 95% confidence interval for analytics (Karl-Fischer-titration, gas chromatography) was smaller than 1%.

**Figure 6:**
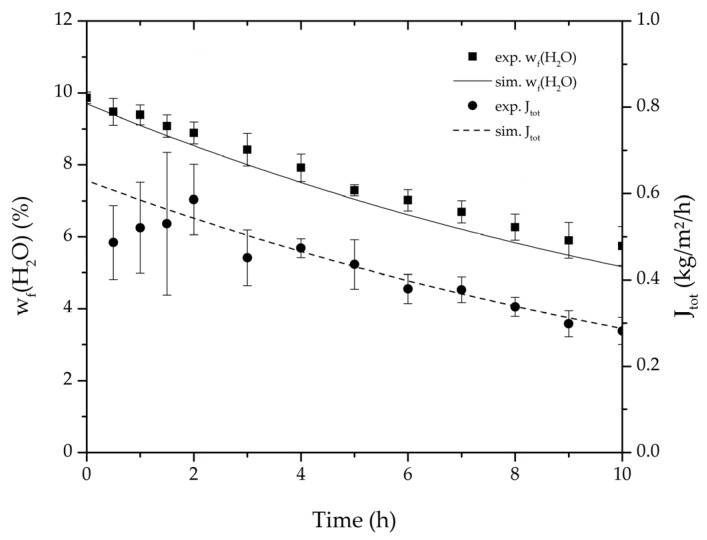
Average water mass fraction of the feed and total permeate flux of the thrice-repeated centre point experiments over the experimental duration for the binary system ethanol/water. The error bars represent the standard deviation, the line the simulation result.

**Figure 7:**
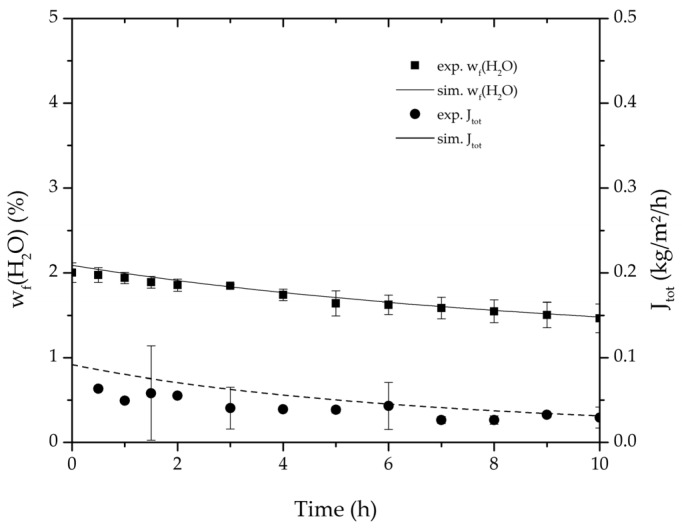
Average water mass fraction of the feed and total permeate flux of the thrice-repeated centre point experiments over the experimental duration for the binary system ethyl acetate/water. The error bars represent the standard deviation, the line the simulation result.

Table 2, Table 3, Table 4 and Table 5 list the dehydration experiments conducted in this study, their respective process conditions and the mixture composition at the end of the experiments. Figure 6 and Figure 7 depict the mean feed mass fraction and total permeate flux over the experimental duration for the centre point experiments of the binary systems ethanol/water and ethyl acetate/water, coupled with the respective error bars representing the standard deviation. The solid and dashed lines represent the respective simulations. Table 6 lists the permeance data determined from the experiments listed in Table 2 and Table 3 for both binary systems. Equation (25) proved to suit in describing the water and ethanol permeance in the binary system. The stronger dependency of the permeance on the water concentration in the feed for the binary system ethyl acetate/water could not be described well with this approach. Thereby Equation (26) was tested and reflected the water and ethyl acetate permeance well. Regarding its physical properties, such as polarity and size, water is closer to ethanol than to ethyl acetate. Hence, the dehydration of the ethyl acetate/water system has a higher selectivity than the dehydration of ethanol. In order to adequately describe these differences in selectivity, different permeance functions can be suitable.

**Figure 8:**
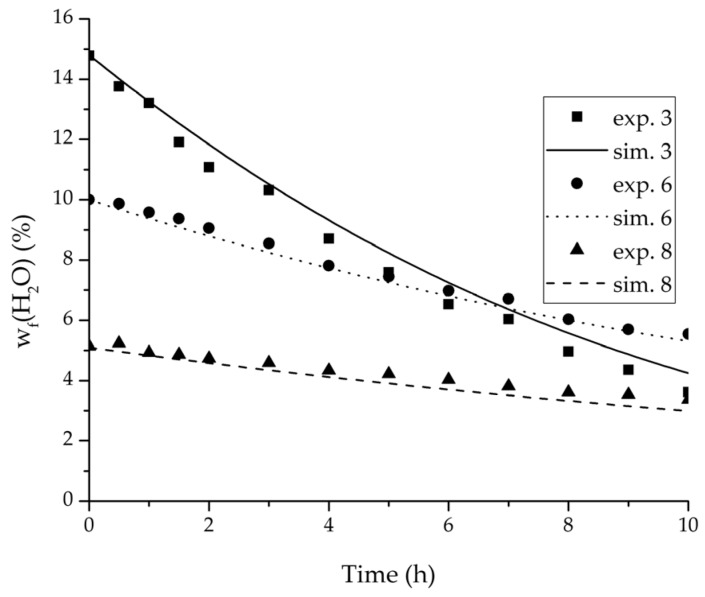
Experimental water mass fractions (feed) over time for three experiments used for the validation of the permeance function for ethanol/water compared with the corresponding simulation result.

**Figure 9:**
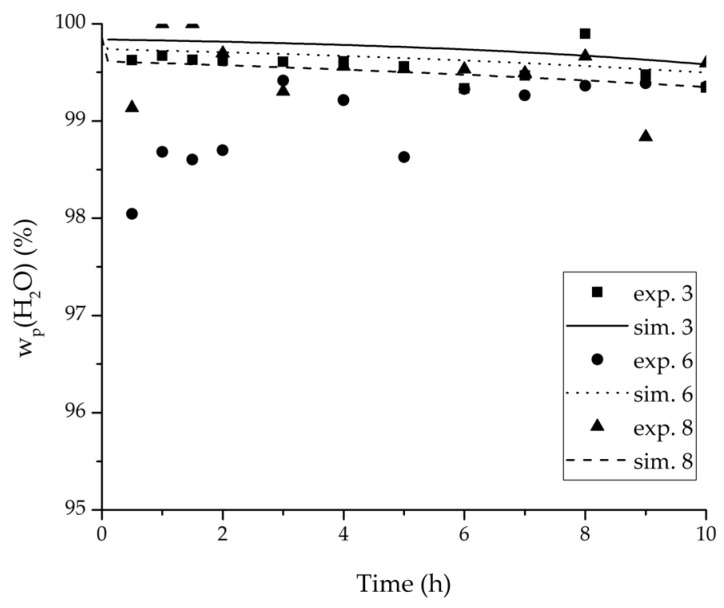
Experimental water mass fractions (permeate) over time for three experiments used for the validation of the permeance function for ethanol/water compared with the corresponding simulation result.

**Figure 10:**
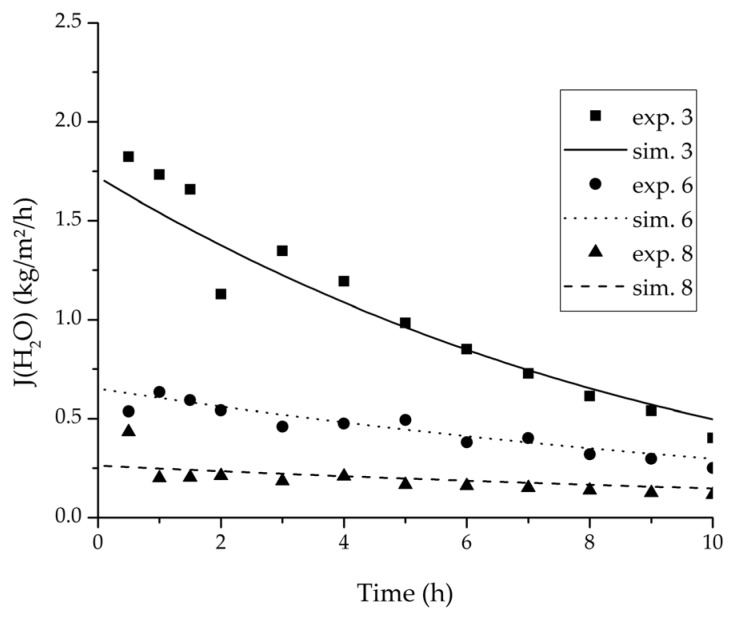
Experimental water flux over time for three experiments used for the validation of the permeance function for ethanol/water compared with the corresponding simulation result.

**Figure 11:**
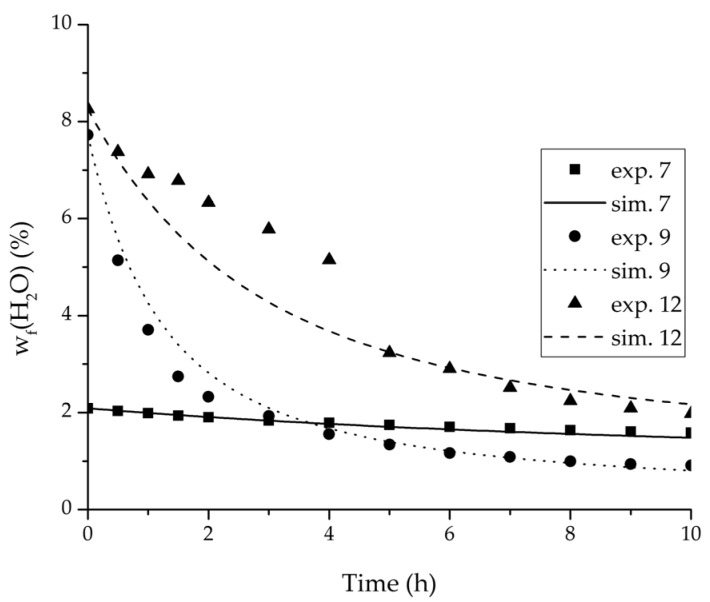
Experimental water mass fractions (feed) over time for three experiments used for the validation of the permeance function for ethyl acetate/water compared with the corresponding simulation result.

**Figure 12:**
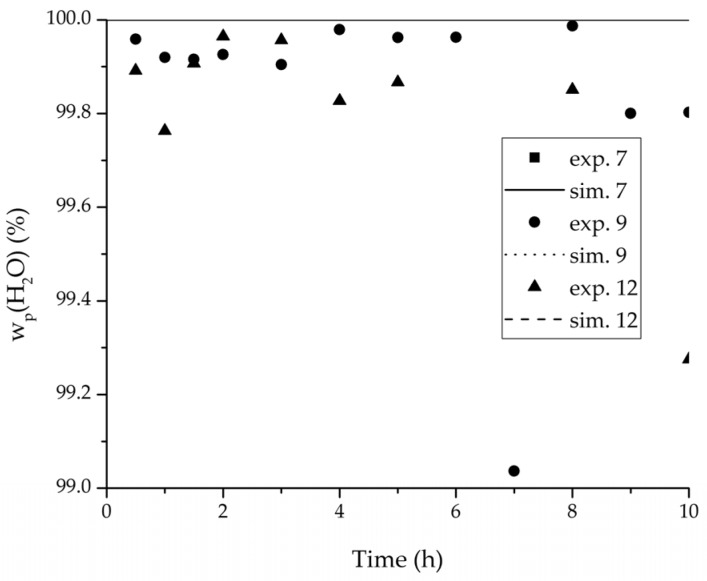
Experimental water mass fractions (permeate) over time for three experiments used for the validation of the permeance function for ethyl acetate/water compared with the corresponding simulation result. All simulation curves are very close to a value of 100%. The flux of exp. 7 was too small to be analysed.

**Figure 13:**
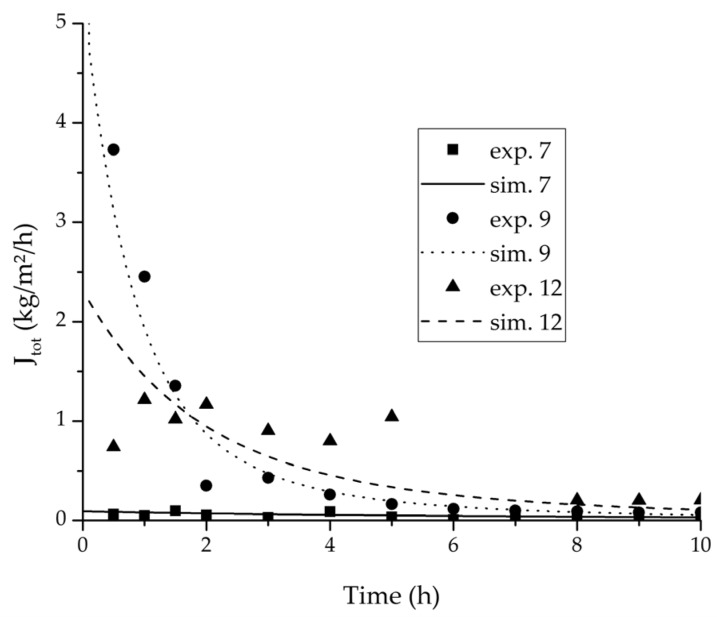
Experimental water flux over time for three experiments used for the validation of the permeance function for ethyl acetate/water compared with the corresponding simulation result.

**Figure 14:**
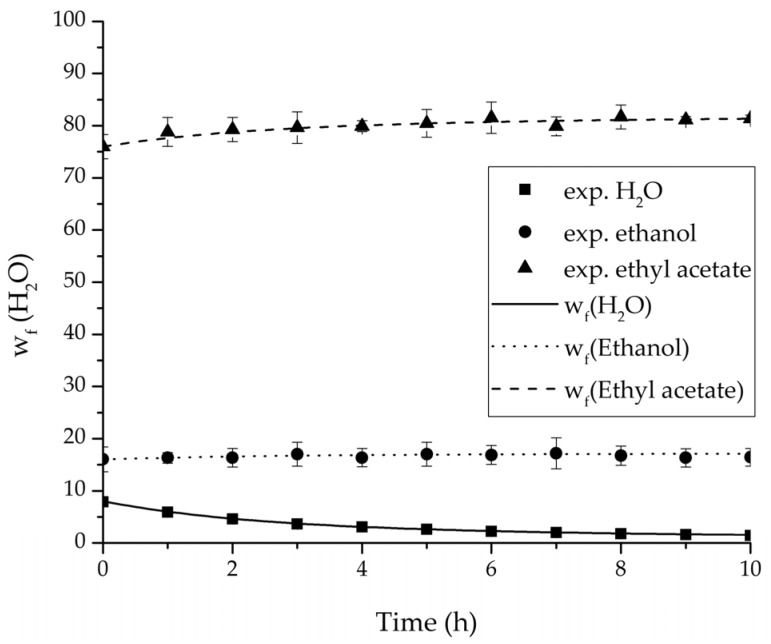
Experimental and simulation feed mass fraction for the ternary system at 95 °C feed temperature.

**Figure 15:**
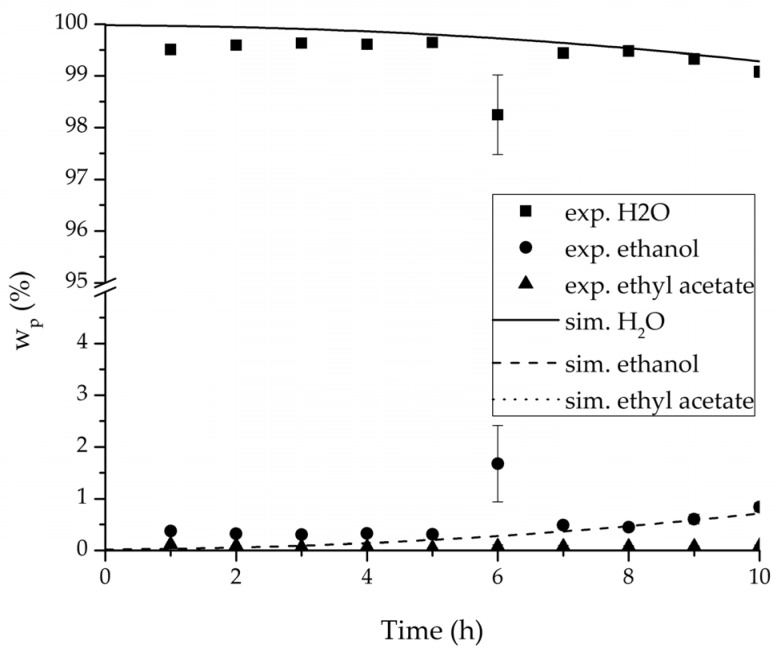
Experimental and simulation permeate mass fractions for the ternary system at 95 °C feed temperature.

**Figure 16:**
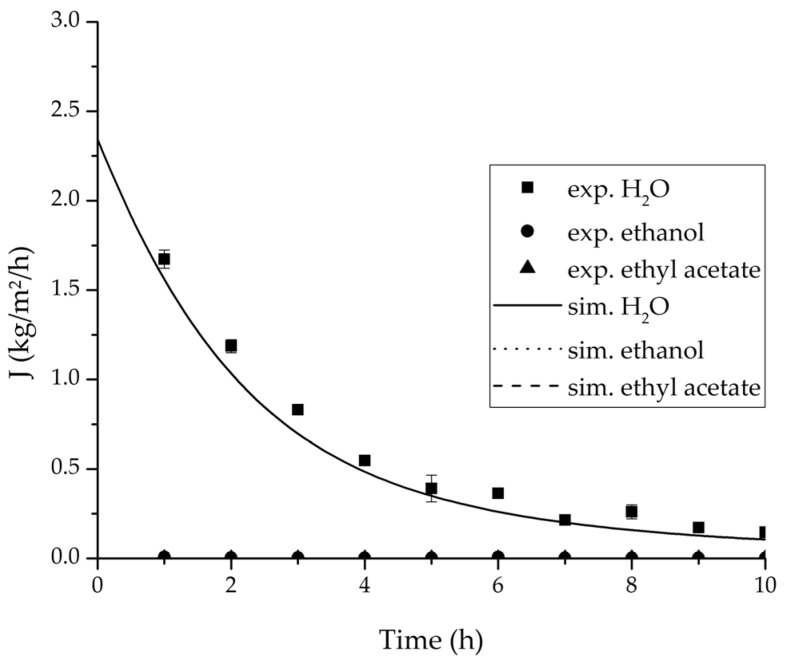
Experimental and simulation permeate fluxes for the ternary system at 95 °C feed temperature.

**Figure 17:**
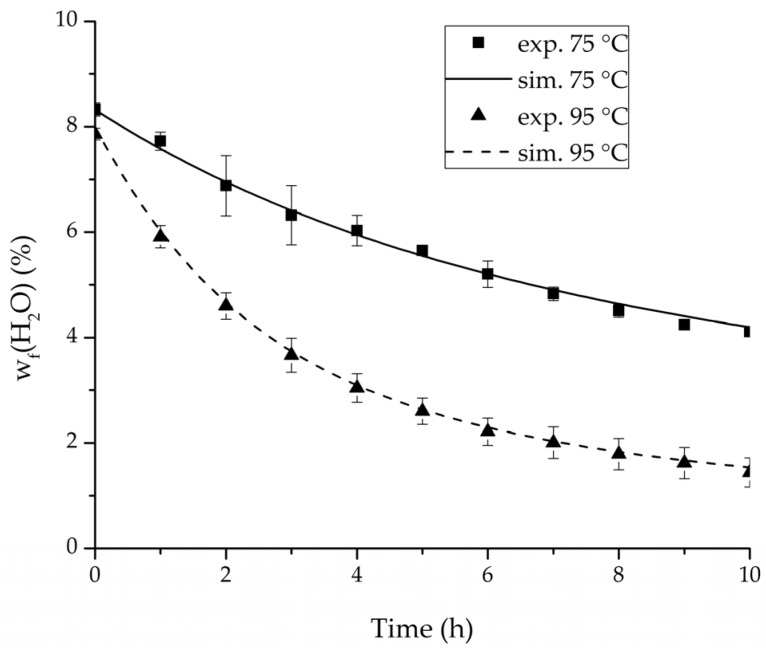
Comparison of the feed water content for the ternary system at 75 °C and 95 °C feed temperature.

**Figure 18:**
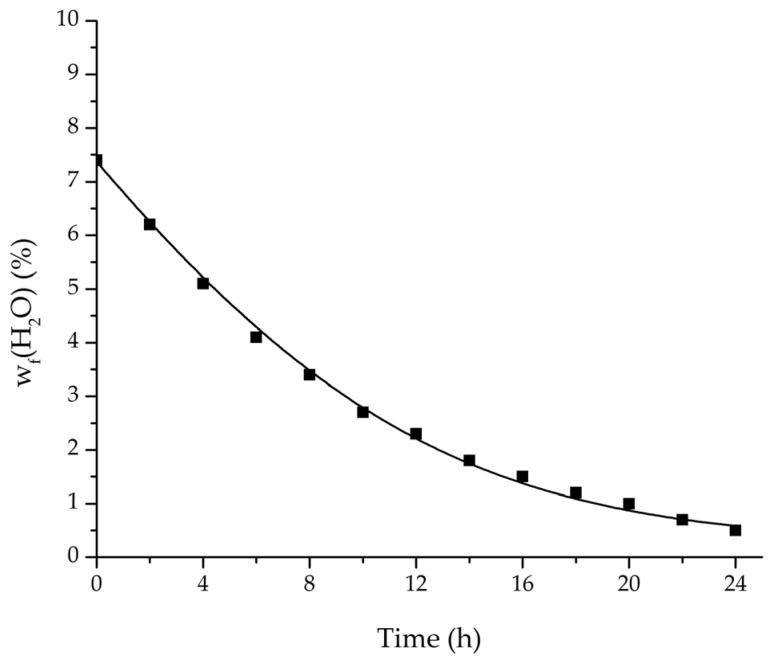
Comparison of the measured and simulated water mass fraction (feed) for the pervaporation plant.

**Figure 19:**
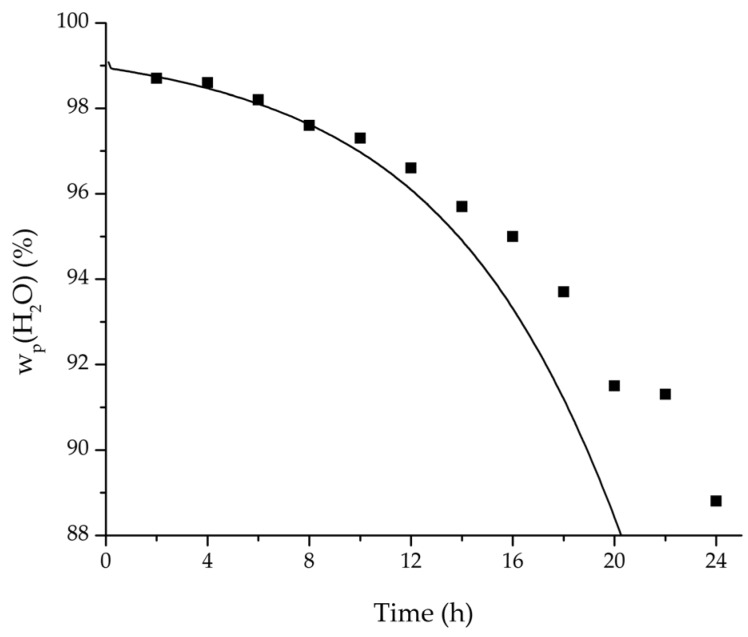
Comparison of the measured and simulated water mass fraction (permeate) for the pervaporation plant.

**Figure 20:**
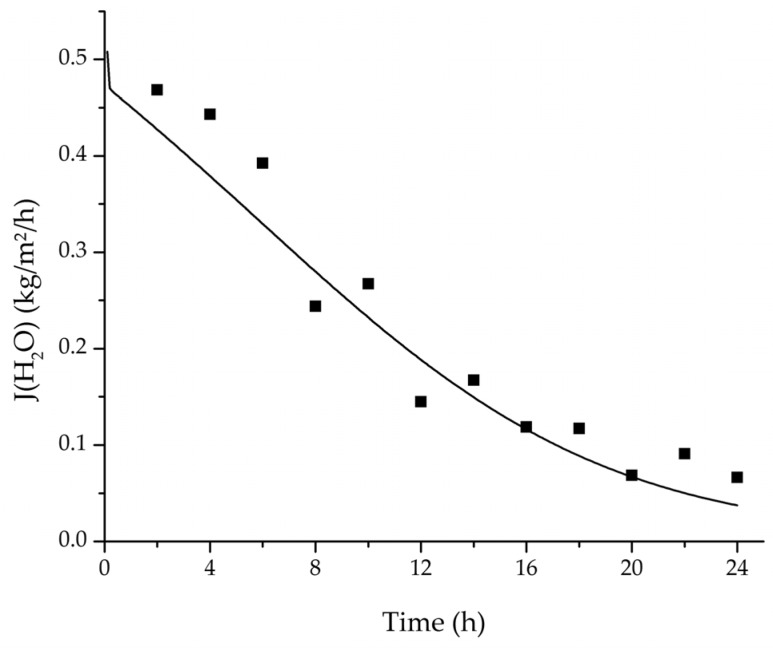
Comparison of the measured and simulated water flux for the pervaporation plant.

In order to validate the physico-chemical model, simulations based on the model coupled with the determined permeance data were run and compared to a second set of binary experiments listed in Table 2 and Table 3 as well. Figure 8, Figure 9 and Figure 10 depict the simulated and experimental water mass fraction of the feed and permeate as well as the water flux over time for three exemplary ethanol/water experiments. Figure 11, Figure 12 and Figure 13 show the equivalent comparisons for three ethyl acetate/water experiments. The simulations of the ternary system were conducted with the permeance data obtained from the binary systems. For ethyl acetate and ethanol, the same correlation and parameters were used to describe the binary and ternary system. To describe the water permeance of the ternary system the water permeance data from the ethyl acetate/water experiments was utilized. Figure 14, Figure 15 and Figure 16 show the mass fraction of feed and permeate as well as the permeate flux of all three components over time for the experiment at 95 °C feed temperature. The average value of the thrice-conducted experiment is thereby compared to the corresponding simulation. The ternary system was investigated for feed temperatures of 95 °C and 75 °C. In Figure 17 the measured and simulated water mass fractions of the feed progression for both temperatures are compared. This comparison is not only of interest as an example of the temperature-dependency of pervaporation processes, but also to test if the simulation reflects this dependency correctly. The scalability of the developed physico-chemical model was investigated by comparing measured large-scale data to a simulation based on the model. Figure 18, Figure 19 and Figure 20 show the water mass fraction of the feed and permeate and the water flux progression over 24 h for process conditions listed in Table 5.

## 5. Discussion

**Figure 21:**
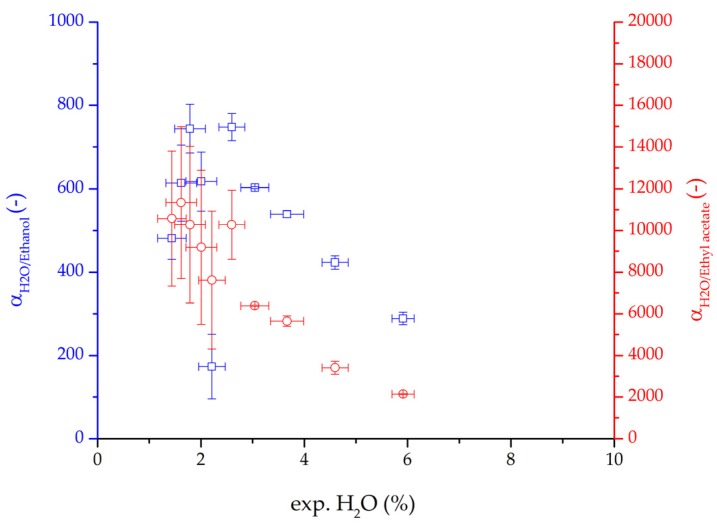
Binary water/ethanol selectivity (blue squares) and water/ethyl acetate selectivity (red circles plotted over the water mass fraction (feed) for the ternary system. The error bars represent the standard deviation.

The focus of this work was the model development and validation. Hence, the variation in feed composition, feed temperature, permeate pressure and feed velocity for both binary systems allowed to test the derived model for a variation of process conditions. This variation also enables the investigation of the influence of these process conditions on key values such as the retentate composition, flux or permeance. Both binary systems showed larger permeate fluxes and permeances for larger water concentrations in the feed. This effect is known in literature as the coupling between water and organic solvent permeances due to the swelling of the polymeric membrane [2,26,27]. Comparing the binary selectivity of water/ethanol to water/ethyl acetate in Figure 21 two characteristics strike the eye. The selectivity of water/ethyl acetate is significantly larger than water/ethanol. Due to the higher polarity of ethanol compared to ethyl acetate this observation was expectable. Both selectivities drop with higher water content, due to the mentioned coupling. The drop in selectivity seems to be proportionally with similar observations made in literature for other solvents [2].

For the varied temperature range a difference in the permeate flux but not in the permeance was detected. The permeance parameter $b_{i}$ is thereby 0 for all components. Hence, the temperature-dependency of the permeate flux is only considered in the physico-chemical model by the driving force. An example for the validity of this approach is seen in Figure 17. The determined permeance data is able to equally describe the water mass fraction in the feed tank for 75 °C as for 95 °C. Another example is, for instance, the shown trajectories of experiments 3, 6 and 8 in Figure 8, Figure 9 and Figure 10 for ethanol/water.

The combination of feed water content and temperature can in each case be very well described. The by theory anticipated influence of the permeate pressure on the permeate flux and thereby the dehydration of the feed mixture was well observed, for example by comparing exp. 10 and 11 in Table 2. Out of the four varied process conditions, the feed volume flow and thereby the feed velocity showed the smallest impact on the permeate flux and mass fractions for the chosen range. 

Figure 8, Figure 9, Figure 10, Figure 11, Figure 12 and Figure 13 illustrate exemplary via the respective comparison between experimental and simulated data the validity of the developed physico-chemical model coupled with the permeance data listed in Table 6. The temperature-independency of the permeance discussed above was again found by the nonlinear regression done for the determination of the permeance data, resulting in the parameter $b_{q,i}$ being 0 for water and both organic solvents. As addressed in Section 2.1.2 a temperature-insensitive permeance or even lower permeances for higher temperatures are possible.

The investigation of the ternary mixture for two temperatures with a feed composition near the industrially-relevant azeotropic mixture revealed two findings. First of all, the temperature-independency of the water and organics permeances was found as well (Figure 17). Second of all utilizing the permeance data of the organic solvents obtained from the binary experiments in the simulation of the ternary system resulted in a very good agreement between simulated and measured data. In case of the water permeances lead only the water permeance parameters obtained by the binary ethyl acetate/water experiments to satisfactory simulation results. Utilization of the discussed permeance data resulted overall in the very good agreement between the experiments and the simulation, illustrated throughout Figure 14, Figure 15 and Figure 16. The deviation of the experimental data at 6 h in Figure 15 is in the author’s opinion an experimental error. The applicability of the binary permeance data for the simulation of the ternary system is a very interesting result. The feed mixture of the ternary experiments contains about 4.75 times the amount of ethyl acetate than ethanol. In the author’s opinion, this is a very probable reason for the better applicability of the water permeance parameters from the ethyl acetate/water data. For a more detailed insight into the permeances of ternary mixtures compared to binary mixtures, an investigation through a detailed experimental design for ternary mixtures incorporating a more profound theory of the sorption and diffusion mechanisms [28,29] would be an appealing topic for future work, but was beyond the scope of this study.

In order to apply the developed physico-chemical model as an engineering tool for the design of pervaporation processes, a proof of principle for the scalability of the model is of key importance. For this reason, large-scale data of a pervaporation unit with 200 m^2^ membrane area was compared to a simulation run with the model. Since the large-scale data was generated for the dehydration of ethanol/water with an identical membrane than in the experiments conducted for this study, the permeance data listed in Table 6 could be transferred. The measured and simulated water concentration in feed and permeate, as well as the permeate flux is shown in Figure 18, Figure 19 and Figure 20. The simulation of the industrial-scale pervaporation unit displays a very good accordance to the measured data. The temperature drops on the feed side of the membrane due to vaporization of the permeating components has a significantly larger effect than temperature polarization. As the key accomplishment of this work the scalability of the developed physico-chemical model combined with laboratory/mini-plant experiments are thereby proven.

**Figure 22:**
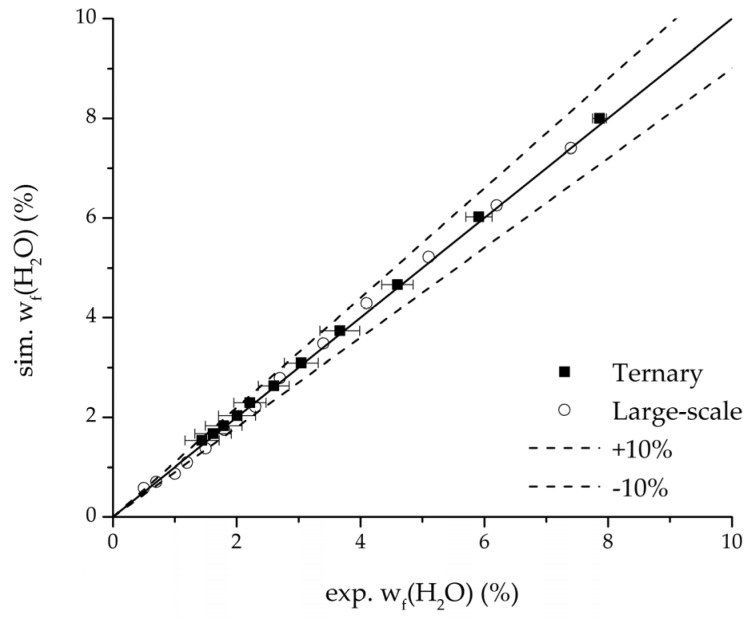
Parity plot between the experimental and simulated water mass fraction (feed) for the ternary system at 95 °C feed temperature.

**Figure 23:**
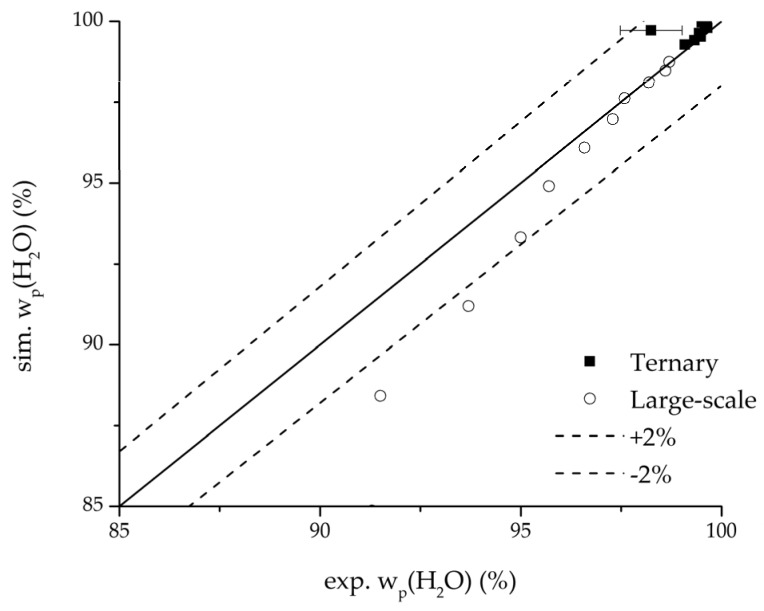
Parity plot between the experimental and simulated water mass fraction (permeate) for the ternary system at 95 °C feed temperature.

**Figure 24:**
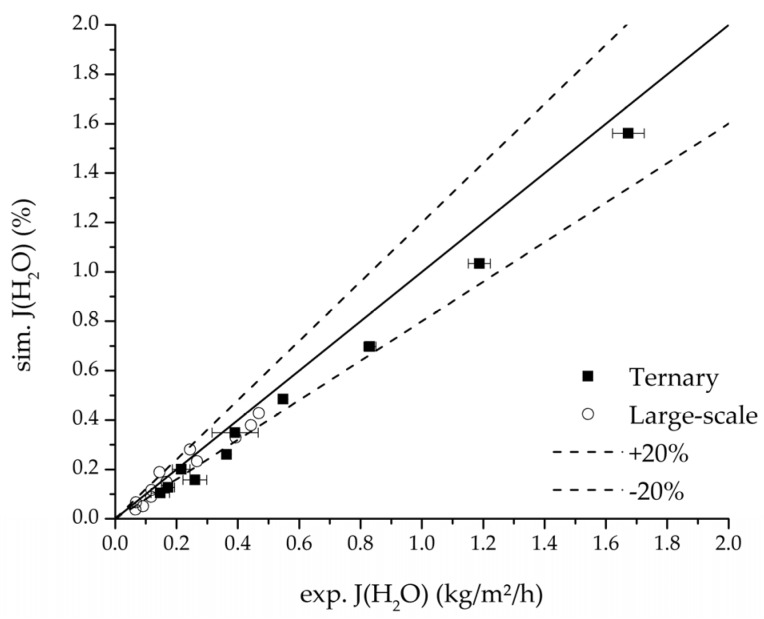
Parity plot between the experimental and simulated water flux for the ternary system at 95 °C feed temperature.

Further, the parity plots between the simulation and measured data displayed in Figure 22 for the water mass fraction in the feed, in Figure 23 for the water mass fraction in the permeate and in Figure 24 for the water flux demonstrate the very good accordance not only for the industrial scale but also for the tested ternary mixture.

## 6. Conclusions

This study shows the development of a physico-chemical model for pervaporation processes. The solution-diffusion mechanism, concentration and temperature polarization, axial dispersion, pressure drop and the temperature gradient due to vaporization of the permeate were implemented into the model. Dehydration experiments with the binary systems ethanol/water and ethyl acetate water were conducted in order to determine the three permeance function parameters needed for each component. Other relevant model parameters were either directly calculated or determined by correlations. The developed model combined with the determined permeance data was validated with a second set of experiments. Dehydration experiments were conducted with the industrially relevant ternary mixture ethanol/ethyl acetate/water. The simulation of the ternary mixture with the permeance data received from the binary experiments (the water permeance was implemented from the ethyl acetate/water data) showed a very good agreement with the experimental data. The scalability of the developed model was investigated by comparing measured data from an industrial-scale pervaporation unit (200 m^2^ membrane area, 15,000 kg ethanol/water feed) with the simulation utilizing the permeance based on the mini-plant experiments. The very good agreement between the measured and simulated permeate flux, feed and permeate composition data proved the scalability of the model. Thereby, the physico-chemical model combined with experiments on a mini-plant scale can be used as an engineering tool for the design of pervaporation processes.

## Figures and Tables

**Table 1:** Parameter sets for Sherwood and Nusselt correlations [21].

Flow Regime	$a_{1}$	$a_{2}$	$a_{3}$	$a_{4}$
Laminar (*Re* < 2300)	1.615	0.33	0.33	0.33
Turbulent (*Re* > 2300)	0.026	0.80	0.30	0

**Table 2:** Experimental design for the binary system ethanol/water. Indicated (*) experiments represent the centre point experiments. Bold highlighted experiments were used for the determination of the permeance, the rest for the validation of the model. All experiments were conducted with feed pressure of 3.5 bar.

Exp.	$w_{f,ethanol}^{start}$ (%)	$T_{f}$ (°C)	$p_{p}$ (mbar)	${\dot{V}}_{f}$ (L/h)	$w_{f,ethanol}^{end}$ (%)
**1**	84.6	55	10	40	84.8
**2**	85.3	75	100	70	87.4
3	85.2	95	10	70	96.4
4	86.2	95	100	40	94.6
**5** *	90.1	85	55	55	93.8
6 *	90.0	85	55	55	94.5
**7** *	90.3	85	55	55	94.7
8	94.9	75	10	70	96.6
**9**	95.0	75	100	40	95.3
10	95.4	95	10	40	98.6
**11**	95.2	95	100	70	97.1

**Table 3:** Experimental design for the binary system ethyl acetate/water. Indicated (*) experiments represent the centre point experiments. Bold highlighted experiments were used for the determination of the permeance, the rest for the validation of the model. All experiments were conducted with feed pressure of 3.5 bar.

Exp.	$w_{f,ethyl\;acetate}^{start}$ (%)	$T_{f}$ (°C)	$p_{p}$ (mbar)	${\dot{V}}_{f}$ (L/h)	$w_{f,ethyl\;acetate}^{end}$ (%)
**1**	97.1	50	10	40	98.2
2	97.2	50	100	70	97.3
3	96.6	70	10	70	97.8
**4**	97.0	70	100	40	98.4
5 *	97.2	60	55	55	97.6
6 *	98.1	60	55	55	98.7
7 *	97.9	60	55	55	98.4
8	99.1	70	10	40	99.1
**9**	92.3	95	10	40	99.1
**10**	93.9	95	100	70	99.0
11	92.5	70	100	40	98.2
12	91.7	70	10	70	98.0

**Table 4:** Overview of process parameters used in the ternary experiments. The mass fractions are given for the start of the experiments and in brackets after 10 h. The mass fractions listed are the mean value based on the thrice-repeated experiments. All experiments were conducted with a feed pressure of 5 bar.

Exp.	$w_{f,ethanol}$ (%)	$w_{f,ethyl\;acetate}$ (%)	$w_{f,H_{2}O}$ (%)	$T_{f}$ (°C)	$p_{p}$ (mbar)	${\dot{V}}_{f}$ (L h^−1^)
1	16.0 (16.4)	76.1 (81.3)	7.9 (1.4)	95	54	70
2	16.0 (17.1)	75.7 (78.1)	8.3 (4.1)	75	54	70

**Table 5:** Process parameters and ethanol mass fraction after 24 h for the industrial pervaporation plant. 15,000 kg of ethanol/water solution were dehydrated, resulting in a total permeate mass of 1070 kg.

$w_{f,ethanol}^{start}$ (%)	$T_{f}$ (°C)	$p_{f}$ (bar)	$p_{p}$ (mbar)	${\dot{V}}_{f}$ (L h^−1^)	$w_{f,ethanol}^{end}$ (%)
92.6	95	6.35	15	8000	99.1

**Table 6:** Determined permeance data for the two binary systems by a nonlinear least squares regression, minimizing the weighted square error between experiments and simulation. For the simulation of the ternary system, the organic solvent permeance data was adopted from the binary data. The water permeance data for the ternary simulation was adopted from the ethyl acetate/water data.

Exp.	Component	$Q_{0}$	$b_{q,i}$	$c_{q,i}$	Equation
binary	ethanol	0.02	0	5	(25)
	water	2.3	0	3	(25)
binary	ethyl acetate	0.01	0	3.1	(26)
	water	361.1	0	3.4	(26)
ternary	ethanol	0.02	0	5	(25)
	ethyl acetate	0.01	0	3.1	(26)
	water	361.1	0	3.4	(26)

## Abbreviations

Symbols		
*A*	*m*^2^	area
$A_{i}$	−	permeance coefficient
a	−	activity
$a_{1}\;to\;a_{4}$	−	*Sh*/*Nu* correlation parameters
$B_{i}$	−	permeance coefficient
c	$\frac{mol}{m^{3}}\;or\;\frac{kg}{m³}$	concentration
${\tilde{c}}_{P}$	$\frac{kJ}{kg\;K}\;or\;\frac{kJ}{kmol\;K}$	specific heat capacity
D	$\frac{m^{2}}{s}$	diffusion coefficient
d	m	diameter
$\dot{H}$	$\frac{kJ}{s}$	enthalpy flow
$\tilde{h}$	$\frac{kJ}{kg}$	specific enthalpy
h	m	height
J	$\frac{kg}{m^{2}h\;}\;or\;\frac{mol}{m^{2}h}$	permeate flux
L	−	proportionality factor
l	m	length
K	$\frac{kg}{m³\cdot bar}$	sorption coefficient
k	m/h	mass transfer coefficient
M	$\frac{kg}{kmol}$	molar mass
m	kg	mass
$\dot{m}$	$\frac{kg}{s}$	mass flow
$\dot{n}$	$\frac{kmol}{s}$	mole flow
Nu	−	Nusselt number
P	$\frac{kg\;m}{m^{2}h\;bar}$	permeability
p	bar	pressure
Pr	−	Prandtl number
Q	$\frac{kg}{m2\;h\;bar}$	permeance
$Q_{i}^{0}$	$\frac{kg}{m2\;h\;bar}$	permeance coefficient
R	$\frac{kJ}{mol\;K}$	gas constant
Re	−	Reynolds number
Sc	−	Schmidt number
Sh	−	Sherwood number
T	K	temperature
u	$\frac{m}{s}$	velocity
$U_{wetted}$	m	wetted perimeter
$\dot{V}$	$\frac{L}{h}$	volume flow
v	$\frac{m³}{mol}$	molar volume
$w_{i}$	$\frac{kg}{kg}$	mass fraction
w	m	width
x	$\frac{kmol}{kmol}$	molar fraction (liquid)
y	$\frac{kmol}{kmol}$	molar fraction (gaseous)
Greek letters		
α	$\frac{J}{h\;K}$	heat transfer coefficient
γ	−	activity coefficient
δ	m	boundary layer thickness
ζ	−	drag coefficient
λ	$\frac{W}{m\;K}$	thermal conductivity
η	Pa⋅s	dynamic viscosity
μ	$\frac{kJ}{mol}$	chemical potential
ν	$\frac{m^{2}}{s}$	kinematic viscosity
ρ	$\frac{kg}{m³}$	density
Subscripts		
b		bulk
ch		channel
Diff		diffusion
eff		effective
f		feed
fm		membrane—feed side
G		gas
h		hydraulic
i,j		components
L		liquid
l		length
m		membrane
p		permeate
pm		membrane—permeate side
Q		cross-section
r		retentate
sat		saturated
tot		total
TM		transmembrane
vap		vaporization
0		reference
